# Optimization Strategy of College Students' Education Management Based on Smart Cloud Platform Teaching

**DOI:** 10.1155/2023/5642142

**Published:** 2023-10-10

**Authors:** Mingjing Zhang

**Affiliations:** College of Teacher Education, Pingdingshan University, Pingdingshan, Henan 467000, China

## Abstract

With the passage of time and social changes, the form of education is also changing step by step. In just a few decades, information technology has developed by leaps and bounds, and digital education has not yet been widely promoted. Intelligent education cloud platforms based on big data, Internet of things, cloud computing, and artificial intelligence have begun to emerge. The research on the “smart campus” cloud platform is conducive to improving the utilization rate of existing hardware equipment in colleges and universities and is conducive in improving the level of teaching software deployment. At the same time, this research also provides a new idea for the research in the field of cloud security. While cloud computing brings convenience to teaching work, it also brings new problems to system security. At present, virtualization technology is still in the ascendant stage in the construction of “smart campus” in colleges and universities and is gradually applied to cloud computing service products. At present, there are many cases about the construction of teaching resource platform, but most of them are modified from the early resource management system, which has strong coupling of single system, insufficient functions of collecting, processing, searching, sharing, and reusing resources, and weak application support ability for related business systems. Under this social background, this paper studies the teaching process management system for intelligent classroom.

## 1. Introduction

In recent years, especially in the last decade or so, with the rapid development and unprecedented prosperity of Internet technology and mobile Internet technology, people's lives have also undergone earth shaking changes [1]. The application of information technology in education and teaching has led to changes in the teaching process, which has attracted the attention of experts, scholars, managers, and front-line teachers. The management of front-line education and instruction, the industrial level, and the nation as a whole have all boosted their information technology efforts. One of the most prominent aspects of the application of information technology in the field of education is the supporting and auxiliary role of teaching resources for daily teaching. Therefore, the construction of teaching resources and the planning of teaching resource service platforms have also become an important part of the educational informatization work [2].

From the multimedia teaching in the late 1990s to today's “smart campus” and “digital campus,” education informatization has experienced a long-term development process. “Cloud computing” technology has been widely used in the field of education since its birth. Based on the advantages of “cloud computing,” higher vocational colleges in China have begun to build cloud platforms, providing a new platform for student management and teaching. At present, most schools have completed the construction of digital campus. Through the digital campus, students can log on to the campus website and do a series of work, such as inquiring about their grades, applying for courses, and paying fees. The digital transformation of the school provides a lot of convenience for students and teachers [3]. However, with the development of cloud technology and information technology and in order to provide students and teachers with a more efficient and intelligent work and learning environment, the transformation from digital campus to smart campus has become an inevitable trend of campus information development [4]. Combined with the bus of school management and teaching, the urgent problems to be solved at present are the classification of freshmen at the beginning of the new school year, the arrangement of courses before teaching, the arrangement of examinations before examinations, and the statistics and analysis of scores after examinations [5]. To solve these problems and realize an efficient, scientific, reasonable, intelligent, and fair intelligent education cloud platform, we need not only programming skills but also rigorous data organization structure and clear and efficient algorithms. Therefore, for the difficult problems in the intelligent education cloud platform, we need to summarize, study, and analyze the relevant algorithms and design algorithms suitable for their respective situations [6].

Cloud computing is a new business delivery model, which completely subverts the traditional concept of terminal management; in a sense, it strips the connection between computer terminal software and hardware and is also a new IT infrastructure management method [7]. As a new computing model, a large number of computer file resource pools are used to carry distributed computing and cloud storage tasks, so as to ensure that different application systems can effectively obtain storage, computing capacity and various software services. The development direction of cloud computing is to improve the cloud computing capacity, effectively reduce the burden of user terminals, and ultimately simplify the user terminals into simple input and output devices, while meeting the user's computing processing needs. Through the new service delivery mode, in the cloud computing mode, users only need to access the network through a thin terminal, and the application software and operating system are provided by the cloud in the background through the network in the form of services, which can make the user terminal fundamental. To avoid multilevel problems such as system paralysis, software conflict, and misoperation [8], smart classroom-oriented learning platform is a form of digital campus, and it is a service platform that provides independent learning for school students and student management for teachers [9]. Because the traditional classroom is only based on the single teaching mode of student-teacher, and there is a lack of communication between students and teachers in the teaching process, teachers cannot know the various states and problems of students in real time in the classroom, and teachers cannot put forward targeted opinions on students' existing problems in time [10]. Introducing the cloud platform into education management can bring new opportunities to colleges and universities, improve the penetration of education work with new methods and concepts, expand the management space, and further optimize the management means. Teachers and students can realize online communication through the cloud platform, help teachers understand students' thoughts, grasp their psychological state and ideological trends, improve the effectiveness of education and management, and make school management work run stably.

The purpose of launching the campus virtual desktop platform based on cloud computing in this paper is to use the virtual desktop technology in cloud computing technology to solve the problems such as the shortage of traditional computers on campus, backward teaching management mechanism, rigid teaching methods, and so on. In short, virtual desktop refers to the technology that supports the remote dynamic access of the desktop system and the unified hosting of the data center at the enterprise level. An image analogy is that now we can access our mail system or network disk on the network through any device, at any place, and at any time. In the future, we can access our personal desktop system on the network at any place and at any time through any device. Virtual desktop technology allows users to install simple thin terminals. Virtual desktop technology allows users to avoid hardware failures of traditional computers; virtual desktop technology can make users free from traditional computer viruses and hacker attacks; virtual desktop technology allows users to avoid cumbersome problems such as software and hardware upgrades of traditional computers. At present, the cloud desktop platform has been widely used in the telecommunications industry and has been gradually extended to the general information system application industry. Virtual desktop technology can save users from hardware failures of traditional computers; virtual desktop technology can save users from viruses and hacker attacks on traditional computers; virtual desktop technology can save users from tedious problems such as software and hardware upgrades of traditional computers. At present, the cloud desktop platform has been widely used in the telecommunications industry and has been gradually extended to the general information system application industry. Compared with traditional teaching resources, modern teaching resources generally have the following characteristics: (1) digitization of resource generation technology, (2) multimedia resource processing methods, (3) network communication of resources, (4) resources are personalized, and (5) resource reuse and sharing.

## 2. Related Work

Since the realization of the strategy for the digitalization of education, China has built the world's largest repository of educational and teaching resources. Over the years, many educational and teaching resources have been developed and constructed.

The construction of teaching resource platform in China is basically synchronized with that in foreign countries. In previous years, due to the great attention paid to the construction of educational resources in China, major resource manufacturers have developed unique teaching resource platforms and sold them to end users as a part of resource services. In recent years, with the introduction of cloud computing technology and the transformation and upgradation of the original resource service platform, domestic resource manufacturers have built a large number of cloud-based resource service platforms to provide users with value-added services.

Reyes and Teaching believed that the distance education system is a manifestation of educational intelligence. In order to improve the design level of this system, virtual reality technology and multiagent system should be fully applied to it. On this basis, scholars constructed the design framework of VR&MAS-DES and clarified the functions of the system, including learning system and student agent, intelligent teaching system, teacher management system, and teacher agent [11]. Alfoudari et al. believed that the combination of virtualization technology and cloud platform has brought a brand-new resource integration and usage model. On-demand resource allocation and scheduling based on virtualization technology can improve the resource utilization rate of cloud platform, improve the quality of cloud services, and reduce the total cost of ownership of cloud users. “Desktop Cloud” is also developing at a high speed, relying on these two technologies. Here is the development status of desktop virtualization, the core technology of desktop cloud [12]. Aguilar et al. believed that the level of cloud computing data transmission protocol design directly affects the satisfaction of cloud computing user experience. The higher the level of design, the higher the efficiency of cloud computing users, the better the user experience, and the higher the user satisfaction and vice versa [13]. Lampolthammer et al. believed that the role of cloud computing in higher education is mainly manifested in the following five aspects: (1) it can reduce the purchase and maintenance costs of school computers and other hardware equipment, (2) it can provide economic application software for schools, (3) it can save energy, (4) it can ensure the information security of teachers and students and improve network security, and (5) it can make data sharing more convenient. In short, the emergence of cloud computing indicates that the current development of the Internet has reached a new stage, which is also a new opportunity [14]. Hou et al. proposed the S0A design of the overall framework of educational administration management. They believed that the advantage of the information system based on SOA is that it has made a more reasonable design for the system architecture, but the shortcomings of C/S mode design are also very prominent: the system is not easy to transplant, the system is not easy to update, and the interface is lack of humanized design [15]. Wolff et al. pointed out that there is a great contradiction between the limited number of class hours arranged by teachers and the students' demand for class selection, the shortage of classroom resources leads to difficulties in class scheduling, the centralized examination at the end of the term leads to tight classroom arrangements, the problem of checking and saving student status information, and the problem of arranging teaching plans [16]. Cheng et al. summed up the multiobjective optimization problem and laid a theoretical foundation. Up to now, some new mechanisms have been introduced into the multiobjective optimization problem, but there are very few articles on the review of the multiconstraint assignment problem, and most of them are an elaboration of NP-hard problems in multiconstraint assignments [17]. Li et al. put forward the SOA design of the overall framework of educational administration and applied cloud computing in the overall framework. Its advantage is that the system architecture is designed reasonably, but the shortcomings of the C/S mode design are also very prominent: the system is not easy to transplant, the system is not easy to update, and the interface lacks humanized design [18]. Campo and Cristina proposed an adaptive queuing algorithm for teaching data preprocessing based on cloud computing to solve the queuing mechanism of educational administration management information system [19–21]. Zhang and Wenjun thought that XenDesktop has the advantage of designing a digital library information management system of Southeast University which can meet the needs of mobile intelligent digital library. The system can realize the key analysis and design of user authentication and secure login of mobile client, inquiry, and online reading of books on mobile devices, urging the return of books on mobile client and paying overdue fines [22–24].

## 3. Methodology

### 3.1. Genetic Algorithm Combined with Cloud Platform Technology Is Used to Analyze Education Management

Since the development of educational informatization, the construction of teaching resources has not been interrupted. At the beginning of the construction of the early campus network, school level resource libraries were equipped for each school, providing some teaching resource content for teachers to use. Cloud computing is a next generation computing model that can provide dynamic resource pooling, virtualization and high availability, and can provide users with “on-demand computing” services. According to the current situation and development trend of education informatization, cloud computing will have extremely important application value in the education industry, such as integrating, developing and utilizing various resources of current education informatization, fully tapping the potential, and improving the utilization rate of resources. Each set of teaching resources is accompanied by a set of resource service platforms, and each set of platforms has its own business logic, data structure, and application system. Jumping and searching between multiple resource platforms to adapt to the operation mode of each platform consume a lot of energy from users, which seriously affect the enthusiasm of users to use, resulting in the phenomenon of not easy to use or unwilling to use. Without a unified construction standard and resource exchange platform, the resource service systems constructed by units at all levels have become isolated islands of resources. However, these resource islands cannot connect well with other business systems, such as lesson preparation system, teaching research system, and self-help learning system, because of their own design problems, and they cannot play a supporting role in basic teaching resources for related business systems. The value of teaching resource service systems built in each stage has shrunk dramatically, and they are gradually forgotten and abandoned, resulting in a large amount of money waste. According to the characteristics of the cloud computing-aided teaching platform, this paper improves the original adaptive genetic algorithm for job scheduling and proposes an improved genetic algorithm to reduce the completion time of tasks and accelerate the response to meet the needs of customers. This algorithm further constrains the selection of genetic genes by adopting the fair mechanism and the data localization mechanism to shorten the total completion time of tasks and improve the satisfaction of users, so as to improve the performance of the algorithm. So that it can better adapt to the cloud computing environment. Based on our school's urgent demand for cloud applications, using XenApp technology, the virtual desktop platform based on cloud computing puts the application execution environment in the cloud in the teaching system and realizes the SaaS service mode that our school strives to build.

**Figure 1:**
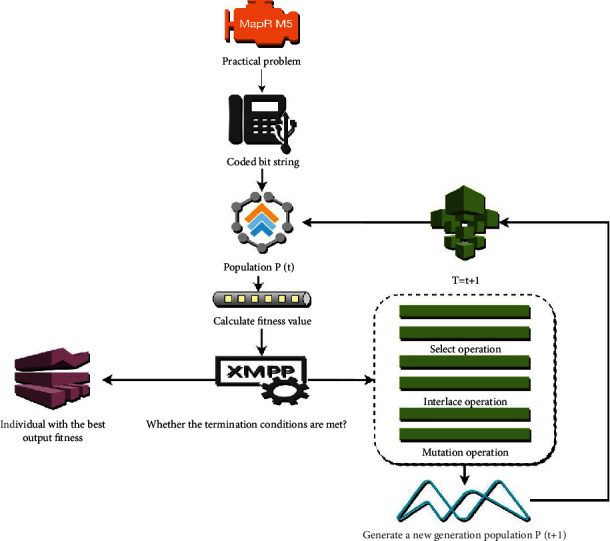
Genetic algorithm process.

The genetic algorithm was first developed by Professor Holland, who put forward stems from his thinking from natural and artificial adaptive systems. The genetic algorithm is a highly parallel, random, and adaptive search algorithm developed by simulating the mechanism of heredity and mutation in the evolution of the biological world. The search mechanism of the genetic algorithm simulates the reproduction, cross-over, and mutation phenomena that occur in the processes of natural selection and natural inheritance. It keeps a set of candidate solutions in each iteration, chooses the better individuals from the candidate solution set in accordance with a certain index, selects them with genetic operators, combines them by cross-over, and mutation, to generate a new generation of candidate solution sets, a process that is repeated until no more candidates are left. It takes “survival of the fittest” as the principle and gradually finds the optimal solution through heredity, mutation, and selection. This is a global optimization strategy, which can avoid falling into local optimization. A combination is randomly selected as the initialization population, and each individual is evaluated one by one from the perspective of finding the optimal solution, and then the fitness can be formed. This value reflects the contribution of these chromosomes to the last problem. According to the initial population, principle of “survival of the fittest,” the genetic evolution is carried out from generation to generation, individuals with low fitness are eliminated, and individuals with high fitness are combined in pairs, so that their excellent genes can be passed on. The genetic algorithm has been widely used in various fields due to its remarkable features such as simplicity, versatility, efficiency, practicability, robustness, and parallelism, and achieved good results, as shown in Figure 1.

If the population size of the genetic algorithm is large, the convergence speed will slow down in the later stage. To solve this problem, the key is to improve the genetic operator and fitness of the genetic algorithm according to the actual situation. The genetic algorithm uses fitness function to calculate and select and evolve the next generation according to the merits and demerits of individuals, so as to find the optimal solution of the problem. Therefore, the selection of fitness function is very important, which will directly affect the convergence speed of the genetic algorithm and the search of optimal solution.

The calculation process of the genetic algorithm is as follows: (1) initialize the population and reasonably set the parameters; (2) through the trained neural network, the excellent value of every individual is judged, and its adaptive value is calculated; and (3) transform the excellent individuals and calculate their fitness values.

### 3.2. Optimization Design of Teaching Management Based on Cloud Computing Platform

The biggest advantage and main feature of cloud computing is resource sharing. Therefore, in the application of cloud computing in university teaching management, we should actively and vigorously promote resource sharing. First of all, we must establish a sound and perfect cloud service platform based on cloud computing. Second, with effective integration of existing cloud platform resources on the teaching management platform based on cloud computing technology, all colleges and universities can upload their own high-quality resources to achieve resource sharing, which can provide valuable knowledge wealth for all higher education teaching, scientific research, and management users on the platform. At the same time, to achieve the most correct effect of resource sharing, these resources are not limited to a region or even a country. They can greatly expand the professional vision of users and master more advanced knowledge.

Cloud computing is a next generation computing model that can provide dynamic resource pooling, virtualization, and high availability and can provide users with “on-demand computing” services. According to the current situation and development trend of education informatization, cloud computing will have an extremely important application value in the education industry. First of all, integrate the software and hardware resources of the teaching parks scattered in different regions; improve their reuse rate; eliminate idleness and waste; achieve unified standards, unified management, and unified maintenance of data; gradually interconnect the data of each branch school and each application system in the campus network dynamically and timely; completely eliminate the information island in education informatization; and realize decentralized information collection, centralized security management, and shared application systems. For example, enterprises provide funds or equipment, universities provide technology and site resources, enterprises provide technology and personnel, and universities provide equipment or site resources. Through good cooperation between schools and enterprises, we can improve our brand value, steadily promote the expansion of computer hardware facilities in ordinary universities, and gradually reduce the hardware facilities restrictions in the development of cloud computing mode. We can promote ordinary colleges and universities to give priority to the service of network cloud platform. With the advantages of cloud platform resources and cloud computing, relatively backward colleges and universities can make up for their shortcomings as soon as possible and achieve the rationality of the distribution of various resources.

Through server virtualization technology, various hardware and software resources are virtualized into one or more resource pools, and these virtual resources are managed and allocated intelligently and automatically through the system management platform. Most private cloud-based computing solutions tend to be IaaS (infrastructure as a service). IaaS mainly includes the following parts: (1) the existing enterprise computing environment is usually an x86 platform, and the “smart campus cloud platform” can be accessed through the server. The virtualization is used to integrate and flexibly utilize computing resources and to integrate, dynamically adjust, and migrate server computing resources. (2) An important part of realizing IaaS is cloud storage. A cloud computing infrastructure needs to serve many different business systems or applications, and each business system or application will have different storage requirements. Through storage virtualization management, the “Smart campus cloud platform” enables integration of storage needs and flexible capacity control. (3) With the large-scale deployment of virtualization technology in the cloud computing environment, the traditional network architecture will face many new challenges, including specifications and performance, virtual machine access and control, large layer 2 network deployment, traffic bursts with congestion, and so on.

**Figure 2:**
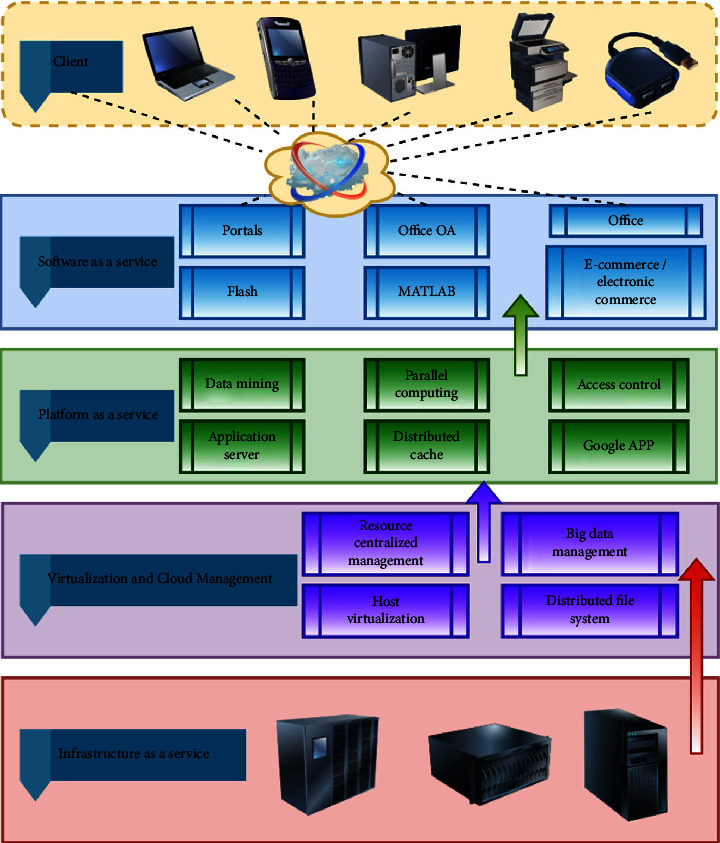
Cloud computing architecture.

“Cloud” has a high performance-to-price ratio. Introducing the cloud computing construction mode to integrate and optimize the private hardware resources in the current information can change the traditional “shaft” IT construction mode and improve the operation efficiency and scalability of the data. At the same time, optimize the way of resource utilization and fundamentally reduce the construction cost of data storage. The cloud computing service architecture is shown in Figure 2.

The infrastructure service layer consists of host, storage, network, and other hardware devices. Virtualization and Cloud management integrates computing resources through virtualization technology and provides basic services such as resources and operating environment to the outside world through pool management. The platform layer mainly provides unified platform system software support services on LAAS, including access control services, data mining, and parallel computing. The application service layer is responsible for external terminal services. Teachers or students can log in to the portal website, cloud application software resources, or virtual desktop according to their needs.

Virtualization technology is the key technology to integrate and utilize various computing resources and storage resources. Among them, server virtualization is to virtualize a physical server into several virtual servers for use. The virtualized entities are all kinds of IT resources, which can realize the deployment of more virtual servers in the limited hardware server environment, so as to reduce the cost of hardware construction, improve resource utilization, and dynamically schedule resources. Traditional virtualization technology, represented by virtual server, is composed of server hardware, server operating system, virtualization software, and virtual machine. Virtual applications rely on virtual operating system. Data are converted from virtual machine to server hardware through three layers. The interfaces, protocols, and communication standards of each layer are different. Therefore, a lot of performance consumption will be generated, resulting in the running speed of virtual machine far behind the real system.

The prediction residual should be processed by discrete cosine transform, the unit is 4 × 4 block, the energy can be concentrated on a few coefficient items in the transform domain, and the spatial redundancy in the video data can be further eliminated. The one-dimensional *N*-point discrete cosine transform can be expressed as

(1) $$\begin{array}{c} y_{k}C_{k}\overset{N-1}{\underset{n=0}{\sum }}x_{n}\cos\frac{\left(2n+1\right)k\pi }{2N}\text{,} \end{array}$$

where *x*_*n*_ is the *n*th item in the input time domain sequence, *y*_*k*_ is the kth item in the output frequency domain sequence, and *C*_*k*_ is defined as follows:

(2) $$\begin{array}{c} C_{k}\left\{\begin{array}{c} \sqrt{\frac{1}{N}}k=0,\\ \sqrt{\frac{2}{N}}k=1,2,...,N-1 \end{array}\right.\text{.} \end{array}$$

The corresponding two-dimensional*N* × *N* discrete cosine transform is carried out as follows:

(3) $$\begin{array}{c} Y_{mn}=C_{m}C_{n}\overset{N-1}{\underset{i=0}{\sum }}\overset{N-1}{\underset{j=0}{\sum }}X_{ij}\cos\frac{\left(2j+1\right)n\pi }{2N}\cos\frac{\left(2i+1\right)m\pi }{2N}\text{,}\\ X_{mn}=\overset{N-1}{\underset{i=0}{\sum }}\overset{N-1}{\underset{j=0}{\sum }}C_{m}C_{n}Y_{mn}\cos\frac{\left(2j+1\right)n\pi }{2N}\cos\frac{\left(2i+1\right)m\pi }{2N}\text{.} \end{array}$$

At the same time, each 4 × 4 sub-block should be taken out from the 16 × 16 luminance block or chrominance block for Hadamard transformation. The encoder will also discard some high-frequency information appropriately, thus reducing the coding length without affecting the video playback effect. The formula for quantizing the conversion coefficient in the matrix *y* is as follows:

(4) $$\begin{array}{c} Z_{ij}=round\left(\frac{Y_{ij}}{Qstep}\right)\text{,} \end{array}$$

where *Y*_*ij*_ is the conversion coefficient of matrix *Y*; *Z*_*ij*_ is the output quantization coefficient; *q* step the quantization step size; quantization parameters are from 1 to 52. For each additional 6 steps, the step will be doubled, and each 12% step will reduce the output encoding rate by approximately 12%. After transformation and quantization, the prediction residuals will be reordered by zigzag, and then the redundancy will be further reduced by fan coding.

It is often said that “customers are God.” To make a platform well, you must stand from the user's point of view and consider everything for the user. Only when you understand what users really need, you can you design corresponding functions according to their needs. The “Smart Education Cloud Platform” is a platform that integrates school management, teacher teaching, student learning, and parental attention. It is aimed at the entire huge education system, ranging from education authorities to children's parents. Therefore, the platform divides the user groups into six user roles: (1) education authorities, (2) school administrators, (3) ordinary teaching teachers, (4) students, (5) parents, and (6) developmental technical service personnel.

One of the main characteristics of the cloud platform is that it can provide personalized services for users and can continuously expand the content and enrich the platform content and modules according to the user's needs. With the help of the cloud platform system management terminal equipment, users can build personalized content and categories according to their own needs and can also realize the remote upgrade of the platform according to their own needs, so as to promote the continuous expansion and docking of cloud platform functions. Teaching management workers are generally users of college teaching management in the cloud platform. They can manage and use the platform according to the development needs of the school and the development needs of teachers and students, provide more professional and targeted services for teachers and students, and realize the personalized development of college teaching management.

In order to reduce the burden of school users and make the platform more convenient to promote, deploy, maintain, and upgrade, the platform adopts B/S architecture. With the development of information technology, more and more software projects put forward higher requirements for packaging, reuse, and relocation. Adopting the B/S mode three-tier architecture is the best way to solve such problems. The Web side adopts Desktop technology based on Exts, and this interface is just like our commonly used windows operating system and mobile app, which not only change users' operating habits but also can load their required functions according to different users. In order to better develop and maintain the page display layer, this layer is divided into three layers according to MVC pattern: data layer, display layer, and control layer. A good database design can help the system improve service efficiency and meet the needs of various system users. The design of the database and the design of the system are synchronous, the requirements of the system are the basis of the database design, and the design of some data is based on the system requirements document. The design purpose of the database system is to effectively and quickly manage a large number of irregular data, store the data according to the rules formulated by the designer, and establish data query index and other information to facilitate data retrieval. The data in the application system may come from the information in the database; it may also be some equipment systems or services, and the database acts as a supporter.

### 3.3. Improved Genetic *K*-Means Clustering Algorithm Model

According to the actual communication between developers and customers, the problem encountered in the teaching system of the virtual desktop platform based on cloud computing is that the continuous growth of existing equipment brings pressure to the computer room space for various departments of the school. The problem encountered in the teaching system of the virtual desktop platform for computing is that the continuous growth of existing equipment brings pressure to the operation and maintenance management of various departments of the school. Schools generally hope to introduce virtual desktop platforms based on heterogeneous cloud computing. The actual requirements of the college's standardized teaching platform based on virtualization and cloud computing technology are as follows: (1) the number of computer room managers in the college is small, generally only one or two people are responsible, so the college requires that the platform must have a good interface. In other words, the management of the platform is required to be simple, which can reduce the workload of managers. (2) The monitoring of the virtual desktop system should be real-time because the number of computer room managers in the college is small (sometimes only one or two people are responsible), so the college data center requires that the standardized teaching platform must be able to monitor all the content of the virtual desktop in real time, and the administrator can extract the computer interface of any operator at any time. (3) In addition to the real-time monitoring module, due to the college's computer room management, the number of personnel is small, and only one or two people are responsible in special cases. Therefore, the data center of the college requires that in the event of a general system intrusion and other dangerous situations, the system can actively send an alarm to the management personnel. (4) The standardized teaching platform also should have intelligent management capabilities. Under the conditions of virtualization and cloud technology, the system can reflect the use of hard disks in an all-round way. (5) The administrators of the college have limited technical level and limited management energy, so they cannot spend a lot of energy in the process of building a standardized teaching platform. It is necessary for the platform construction procedure to be straightforward and uncomplicated, with clear usage and system construction instructions, and a preplanned handling mechanism for frequently encountered common problems, which can form a detailed standardized teaching based on virtualization and cloud computing technology. The *k*-means algorithm assumes that there is no change in the adjacent cluster centers, the data object adjustment is completely completed, and the clustering criterion function *J* converges as the termination condition. A feature of the algorithm is that each data object is checked for correct classification during each iteration, and if not, it is reassigned. After all the data are allocated, modify the cluster center and objective function value and enter the next iteration. The *k*-means algorithm usually uses Euclidean distance as an index to measure similarity, and the objective function *J* for evaluating the quality of division can be defined as

(5) $$\begin{array}{c} J=\overset{k}{\underset{i=1}{\sum }}\overset{n}{\underset{j=1}{\sum }}w_{ij}d_{ij}\text{,} \end{array}$$

where *k* is the number of classes, *n* is the total number of sample points, *d*_*ij*_ = ‖*x*_*i*_ − *z*_*i*_‖ is the Euclidean distance, which indicates the distance from the original sample point *x*_*j*_ to the center *c*_*i*_ of class *z*_*i*_, and *z*_*i*_ is the average of all data objects in class *c*_*i*_, indicating which class the data objects belong to.

(6) $$\begin{array}{c} w_{ij}=\left\{\begin{array}{c} 1\text{,}\\ 0\text{.} \end{array}\right. \end{array}$$

Formula (5) can also be expressed as

(7) $$\begin{array}{c} J={\overset{k}{\underset{i=1}{\sum }}\underset{x_{j}\in C_{i}}{\sum }d_{ij}}^{2}\text{.} \end{array}$$

*d*_*ij*_^2^ in this formula refers to the sum of the squared errors between the data object and the corresponding cluster center, so the objective function is also called the error sum of squares criterion function. Calculate the distance between each remaining data object and the cluster center, if it is satisfied.

(8) $$\begin{array}{c} D\left(x_{i},c_{k}\right)=\min\left\{D\left(x_{i},c_{j}\right),i=1,2,3,\cdots i=1,2,3,\cdots n,j=1,2,3,\cdots k\right\}\text{,} \end{array}$$

where *x*_*i*_ is classified into class *C*_*k*_. According to the points in the divided sets, a new cluster center *c*_1_^*∗*^, *c*_2_^*∗*^, *c*_3_^*∗*^, ⋯ , *c*_*k*_^*∗*^ is calculated as follows:

(9) $$\begin{array}{c} c_{j}^{*}=\frac{1}{n_{j}}\underset{x_{m}\in C_{j}}{\sum }x_{m}j=1,2,3,\cdots K\text{,} \end{array}$$

where *n*_*j*_ is the number of points in class *C*_*j*_. Given the data object set *X* = {*X*1 (1, 1), *X*2 (1.2, 1.2), *X*3 (0.8, 1.2), *X*4 (0.9, 0.7), *X*5 (1.3, 0.9), *X*6 (1, 1.4), *X*7 (3, 3), *X*8 (3.1, 2.8), *X*9 (3.2, 3.4), *X*10 (2.7, 3), and *X*11 (2.6, 2.9)}, the number of categories *k* = 2.

THE first iteration: select the third data object (0.8, 1.2) and the eighth data object (3.1, 2.8) as the initial cluster centers of classes *C*1 and *C*2. Calculate the distance from the first data object to the two clustering centers:

(10) $$\begin{array}{c} \left\|x_{1}-x_{3}\right\|=\sqrt{{\left(1-0.8\right)}^{2}+{\left(1-1.2\right)}^{2}}=0.283\text{,}\\ \left\|x_{1}-x_{3}\right\|=\sqrt{{\left(1-3.1\right)}^{2}+{\left(1-2.8\right)}^{2}}=2.766\text{.} \end{array}$$

It can be seen from the abovementioned equations that ‖*x*_1_ − *x*_3_‖ < ‖*x*_1_ − *x*_8_‖ divides *x*1 into the class to which *x*3 belongs.

According to the data objects in the divided classes, recalculate the cluster center of each class:

(11) $$\begin{array}{c} z_{1}=\frac{\left(\left(1+1.2+0.8+0.9+1.3+1\right),\left(1+1.2+1.2+0.7+0.9+1.4\right)\right)}{6}=\left(1.033,1.067\right)\text{.} \end{array}$$

The second iteration: using *Z* (1.033, 1.067) and *Z* (2.92, 3.08) as the clustering center of class *C*, redivide the dataset.

*X*_1_, *X*_2_,*X*_3_, *X*_4_, *X*_5_, *andX*_6_ are divided into class *C* to which *x* belongs, and *X*_1_*X*_2_*X*_3_*X*_4_ are divided into class *c* to which *x* belongs.

(12) $$\begin{array}{c} Z_{1}=\left(1.033,1.067\right)=z_{1}\text{,}\\ Z_{2}=\left(2.92,3.08\right)=z_{2}\text{.} \end{array}$$

During the two iterations, if the data objects in the two classes are changed, the iteration process is stopped. The two clusters obtained are ={*X*_1_, *X*_2_, *X*_3_, *X*_4_, *X*_5_,} and *c*={*X*_1_, *X*_2_, *X*_3_, *X*_4_}.

## 4. Result Analysis and Discussion

Conceptual structure design is the first step of the database design. Conceptual design is a necessary condition for the successful design of logical database, which affects the design of the whole database. The data model includes two convenient contents. On the one hand, it is the static characteristics of data: it mainly includes the basic structure of data, the relationship between data, and the mutual constraints between data; the other is the dynamic characteristics of data: mainly including the methods of data operation. In the conceptual design of data, the commonly used method is to use the entity relation design model. After collecting and processing users' information and analyzing their needs, we provide users with personalized recommendation services. As the last link of the system, the selection and call of the teaching process service play a vital role in the final success or failure of the system. The development of this research system needs two parts: hardware environment and software environment. The hardware needed is router, reader, tag, computer, and network cable; the software environment used is win7 platform, and the program development language used is java programming language. The specific development, operation, and network environment of the system are shown in Table 1.

**Figure 3:**
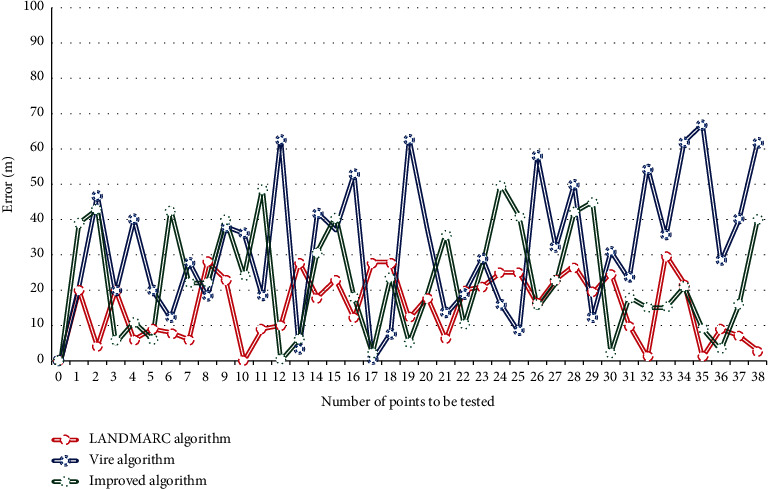
Positioning errors of different algorithms.

**Figure 4:**
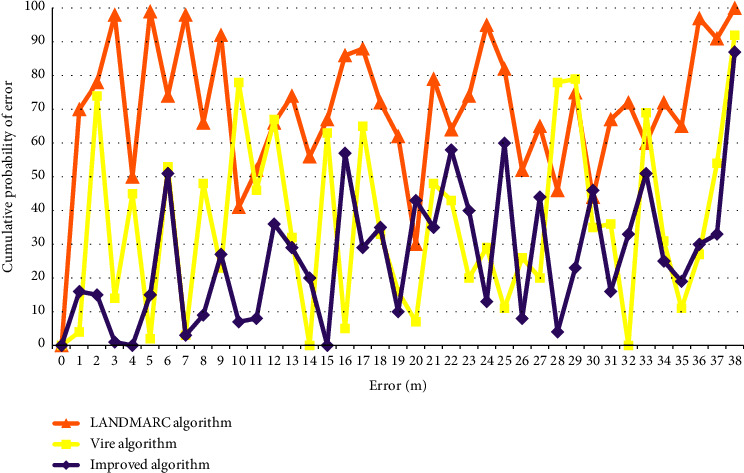
Error CDF curves after fitting by different algorithms.

Selection, also known as heredity, is a natural law that simulates “survival of the fittest” in the biological world. For the initial population, not all individuals are scientifically reasonable, so it is necessary to select excellent individuals to pass on good genes to the next generation, so that the good varieties can be continued, and one generation is better than the other. There are many ways, such as gambling method and sorting method, to choose. This article will use the gambling method to select chromosomes for inheritance. When acquiring the location context information, the outdoor location context information is obtained through the GPS sensor that comes with android. There is a class Location Manager in Android that can locate the location of the mobile phone. For indoor location context information, we use RFID to determine the specific location of the student, and the specific method used is the improved VIRE algorithm mentioned in the previous section. Figures 3 and 4 are the comparison of the positioning error of the improved VIRE algorithm with the original VIRE algorithm and LANDMARC algorithm, as shown in Figures 3 and 4.

The gambling method is equivalent to placing a pointer in the picture below to rotate randomly and selecting chromosomes as parents from which area you go. In order to ensure “survival of the fittest,” each area of the gambling table represents a certain range of fitness, and the population with high fitness occupies a large area, while the population with low fitness occupies a small area.

From the actual demand analysis of the virtual desktop teaching platform based on cloud computing, the system involves the following roles, as shown in Table 2.

**Figure 5:**
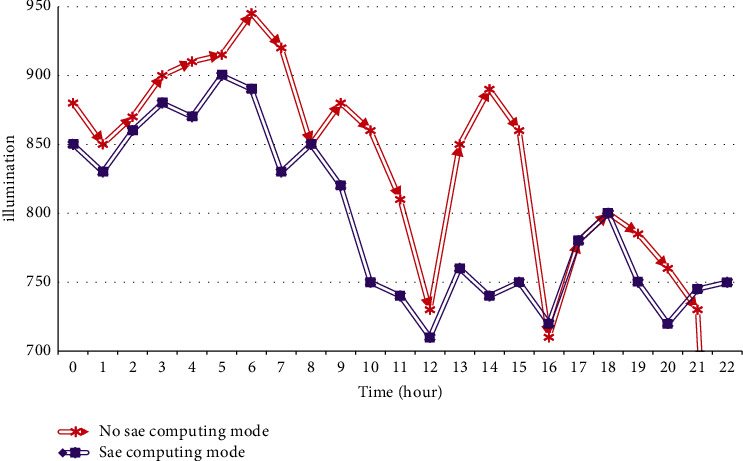
Comparison of teachers' lighting.

**Figure 6:**
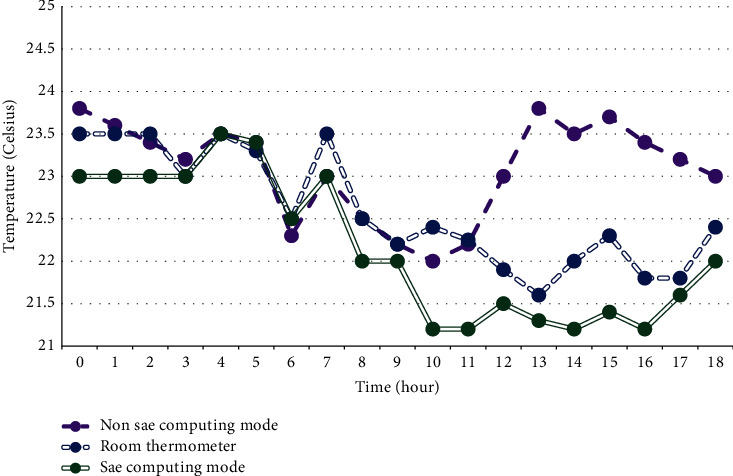
Temperature reading experiment diagram.

A good physical classroom environment can effectively promote the teaching activities and directly affect the physical and mental activities of teachers and students. Combined with the characteristics of the Internet of Things technology, real-time comprehensive detection of changes in the classroom environment can be carried out. The experiment selects two factors that have a greater impact on teaching activities, namely, classroom temperature and light conditions, as the experimental monitoring objects, and uses two sets of different sensors to monitor the temperature and light changes in the selected classrooms to test the stability of intelligent nodes.  Figure 5 shows the changes in lighting in the smart classroom, and Figure 6 shows the changes in indoor temperature. Figures 5 and 6.

As shown in Figures 5 and 6, the data monitored by different sensors in the same classroom area, the same deployment position, and the same working time have no obvious difference within the allowable error range due to their own energy consumption and the existence of heat generation problems of the equipment.

With the advent of the Internet era, the number of users has increased dramatically, and the performance of the website system has become an important indicator of the quality of the system. The performance indicators of the system mainly include response speed, the maximum number of concurrent users, and the maximum number of online users. After the development of this system, test tools are used to fake user interaction and send requests to the server concurrently.  Table 3 shows the test cases of added functions.

**Figure 7:**
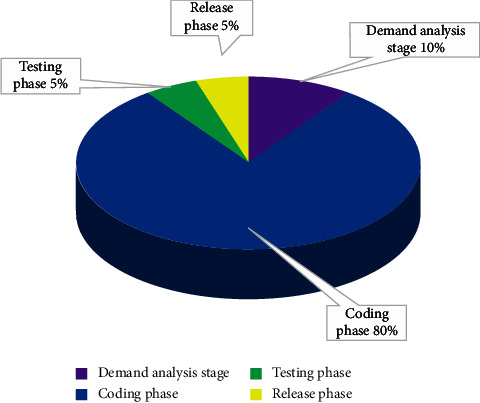
Analysis of test bug introduction stage results.

This section mainly analyzes the bug introduction stage and defect introduction stage in detail. As shown in Figure 7, the result analysis of the test bug introduction stage is to analyze the proportion of the system requirements' analysis stage, coding stage, guessing stage, and release stage, respectively. It can be seen that most bugs are introduced in the coding part. It is necessary to strengthen the unit test of the system and realize the automation of the unit test. The number of bugs found in the release phase and testing phase is low, as shown in Figure 7.

**Figure 8:**
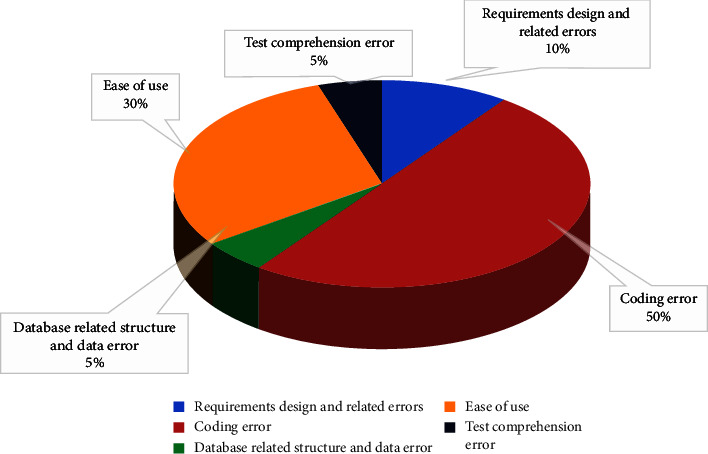
Distribution of defect introduction causes.

As shown in Figure 8, the distribution diagram of defect introduction causes is analyzed in three parts: requirement design and related errors, coding errors, data corresponding results and data errors, ease of use, and test understanding errors, as shown in Figure 8.

The whole test results can be summarized as follows: (1) the biggest problem in the test process is the variability of requirements. Due to the deviation of developers and testers' understanding of requirements, everyone works with their own understanding. At this time, some bugs will be generated in the process of work. In addition, the requirements are not accurately defined, and these requirements are related to other functions. The requirements are changed, and the development and testing are also changed. (2) This test is mainly due to manual testing, and it cannot realize a large number of data operation function tests.

## 5. Conclusions

Aiming at the research topic of the teaching process management system for smart classroom, this paper is divided into five parts to analyze and implement. First, it provides convenient resource push and resource support services for the collaborative lesson preparation module, network teaching and research module, and teacher training module and other applications in the regional education cloud platform, which has achieved the original intention of the design. At the same time, because of the cloud computing architecture, it guarantees efficient and powerful computing power and its own stability. Second, the application set of smart campus is studied, and the construction scheme, demand analysis, and system design of the application set are analyzed in detail, so as to complete the construction of resource sharing platform, teaching platform, and simulation system. This paper provides a test scheme for the application platform of the smart campus, gives the scheme of function test and performance test, and makes a test summary. It provides reference for the construction of similar educational service-sharing platforms. This paper mainly studies the method of building a smart campus cloud service platform and carries out demand analysis, design and implementation, and test summary. The paper is mainly divided into two parts: cloud computing service platform and cloud application platform.

## Figures and Tables

**Table 1:** Development environment.

	Client	Server side
Development environment	Myeclipse	MyEclipse + Protege + mysql
Operating environment	Android4.4	Win7 + Tomcat
Network environment	WLAN + GPRS	TYUT_IPv6

**Table 2:** Role table.

Role	Responsibility or function
Platform management personnel	The main participants of platform management are responsible for the daily hardware maintenance and original update of the platform
Student	Main participants of the platform and users of various tasks of the platform
Teacher	Main participants of the platform and users of various tasks of the platform
Supermanager	The main participants of the platform are responsible for the overall management of the platform

**Table 3:** Added test cases of course information input.

Test item	Added course information
Test content	Enter everything and click the “Submit” button
Preset condition	Turn on the server
Submission time	16 : 2 : 18
Reaction time	16 : 2 : 18
Time spent	<1 second
Print completion time	21 seconds
Time spent	1 second

## Data Availability

The figures and tables used to support the findings of this study are included within the article.
